# The *Legionella pneumophila* Effector VipA Is an Actin Nucleator That Alters Host Cell Organelle Trafficking

**DOI:** 10.1371/journal.ppat.1002546

**Published:** 2012-02-23

**Authors:** Irina Saraiva Franco, Nadim Shohdy, Howard A. Shuman

**Affiliations:** 1 Department of Microbiology and Immunology, Columbia University Medical Center, New York, New York, United States of America; 2 Department of Microbiology, University of Chicago, Cummings Life Science Center, Chicago, Illinois, United States of America; The Rockefeller University, United States of America

## Abstract

*Legionella pneumophila*, the causative agent of Legionnaires' disease, invades and replicates within macrophages and protozoan cells inside a vacuole. The type IVB Icm/Dot secretion system is necessary for the translocation of effector proteins that modulate vesicle trafficking pathways in the host cell, thus avoiding phagosome-lysosome fusion. The Legionella VipA effector was previously identified by its ability to interfere with organelle trafficking in the Multivesicular Body (MVB) pathway when ectopically expressed in yeast. In this study, we show that VipA binds actin *in vitro* and directly polymerizes microfilaments without the requirement of additional proteins, displaying properties distinct from other bacterial actin nucleators. Microscopy studies revealed that fluorescently tagged VipA variants localize to puncta in eukaryotic cells. In yeast these puncta are associated with actin-rich regions and components of the Multivesicular Body pathway such as endosomes and the MVB-associated protein Bro1. During macrophage infection, native translocated VipA associated with actin patches and early endosomes. When ectopically expressed in mammalian cells, VipA-GFP displayed a similar distribution ruling out the requirement of additional effectors for binding to its eukaryotic targets. Interestingly, a mutant form of VipA, VipA-1, that does not interfere with organelle trafficking is also defective in actin binding as well as association with early endosomes and shows a homogeneous cytosolic localization. These results show that the ability of VipA to bind actin is related to its association with a specific subcellular location as well as its role in modulating organelle trafficking pathways. VipA constitutes a novel type of actin nucleator that may contribute to the intracellular lifestyle of Legionella by altering cytoskeleton dynamics to target host cell pathways.

## Author Summary

*Legionella pneumophila* is a facultative intracellular bacterium that can cause an often fatal type of pneumonia known as Legionnaires' disease. In nature, *L. pneumophila* is found in both fresh water and soil where it parasitizes free-living protists. Upon inhalation of contaminated aerosols, *L. pneumophila* invades and replicates in alveolar macrophages, leading to inflammation and development of the disease. Legionella uses a type IVB secretion system to translocate effector proteins into the host cell that modify its trafficking pathways and prevent fusion of the newly formed phagosome with the lysosome. One of these effectors is VipA, which, when expressed in yeast interferes with the Multivesicular Body (MVB) pathway. We found that VipA protein binds actin and nucleates its polymerization without additional host factors. VipA localizes in puncta in eukaryotic cells, and these colocalize with actin-rich regions and endosomes. We demonstrate that the ability to disrupt the MVB is associated with the capacity to bind actin. Thus VipA may contribute to the intracellular lifestyle of *L. pneumophila* by targeting the cytoskeleton in order to disrupt normal vacuolar trafficking pathways in host cells.

## Introduction

The gram-negative bacterium *Legionella pneumophila* is the causative agent of a severe type of pneumonia known as Legionnaires' disease [1]. Infection of mammalian alveolar macrophages is believed to be largely accidental and occurs after inhalation of aerosols originating from contaminated water sources, where the intracellular pathogen thrives within its natural protozoan hosts. After phagocytosis, Legionella replicates in a specialized vacuole that avoids the endocytic pathway and supports bacterial replication [2]–[4]. The type IVB Icm/Dot (Intracellular Multiplication/Defective Organelle Trafficking) translocation system is essential for the processes that prevent the phago-lysosome fusion [5]–[11]. Effector proteins injected into the host cell by the Icm/Dot T4BSS are presumed to modify trafficking pathways thus avoiding bacterial degradation and promoting the formation of a replication-competent Legionella-containing vacuole (LCV). Although to date approximately 300 Icm/Dot substrates have been identified, the functions of most effectors remain unknown. With few exceptions, the absence of one effector does not impair intracellular growth, an occurrence believed to be due to functional redundancy among effectors and/or host cell targets proteins or pathways. In fact, a recent study has demonstrated that a simultaneous deletion of 31% of known Legionella Type IVB substrates minimally affected intracellular growth in mouse macrophages [12]. Therefore, classical forward and reverse bacterial genetics have been mostly ineffective in determining the functions and contributions of effectors to intracellular events during infection. Thus alternative approaches such as bioinformatics and biochemistry have been used to elucidate the roles of translocated effectors in a small number of cases.

The conserved organelle trafficking pathways between *Saccharomyces cerevisiae* and higher eukaryotes, its amenability for genetic manipulation and extensive library of mutants and strains expressing fluorescently-tagged proteins make yeast an attractive model for studying the functions of pathogen effectors. A successful strategy relied on the ectopic expression of Legionella genes in *S. cerevisiae* and screening for mistrafficking of proteins to the vacuole (Vacuolar Protein Sorting/VPS). One of the Legionella effectors leading to a Vps^−^ phenotype is the VipA protein (VPS inhibitor protein A). We previously showed that in yeast the VipA-derived interference in organelle trafficking was related to the Multivesicular Body (MVB) Pathway, and that VipA is a *bona fide* Icm/Dot substrate translocated into macrophages [13].

In this work we sought to functionally characterize VipA using *in vivo* and *in vitro* approaches. We found that VipA binds actin and directly enhances its polymerization *in vitro*. During infection of macrophages, translocated VipA localizes in punctate structures that associate with filamentous actin and early endosomes. Similarly, hybrid VipA-GFP and VipA-mCherry localize in puncta when ectopically expressed in both mammalian and yeast cells. In budding yeast the puncta are often associated with the vacuole membrane, reminiscent of the pre-vacuolar compartment seen in *vpsE* mutants defective for MVB formation, and co-localize with actin-rich structures/organelles, such as the bud-neck, endosomes or cortical actin patches. In mammalian cells, VipA-EGFP puncta co-localize with early endosomes and actin patches, but not other components of the endosomal or secretory pathways. In contrast, a VipA mutant (VipA-1) that no longer causes a Vps^−^ phenotype in yeast displays a homogeneous cytosolic distribution and is defective in actin binding *in vitro*.

These results show that Legionella effector VipA associates with components of the endocytic pathway and that this function is linked to its modulation of actin dynamics, suggesting a mechanism of interference with host cell organelle trafficking pathways during infection.

## Materials and Methods

### Strains and media

*L. pneumophila, S. cerevisiae* and *E. coli* strains used in this work are listed in Table S1. Bacterial strains were grown as previously described [14]. Deletion of *vipA* in strain LPIF3 was generated by natural transformation of KS79 [15] with a DNA fragment containing PCR products of a kanamycin resistance cassette and flanking regions of *vipA*. The PCR products were synthesized by long-flanking homology PCR [16] using primers IF04, IF05, IF06 and IF07 (see Table S2).

### Plasmid construction

Plasmids and oligonucleotides used in this study are listed in Table S2. For expression of GFP tagged proteins in mammalian cells, plasmids pIF203 and pIF213 were constructed. PCR amplification of *vipA* and *vipA-1* mutant was carried out using as template chromosomal DNA from strain JR32 and plasmid DNA pET15b-vipA-1, respectively. Oligonucleotides IF02 and IF03 allowed the introduction of an XhoI site and a Kozak consensus sequence immediately upstream the 5′ end of the gene, and a BamHI site at the 3′end. PCR products were sub-cloned at the same sites of pEGFP-N1 (Clontech) generating translational fusions of the *vipA* alleles to EGFP under the transcriptional control of the CMV I/E promoter.

For expression of GFP fusion proteins in yeast, plasmids pIF206 and pIF209 were generated by PCR amplification of *vipA* and *vipA-1*, respectively, using the same templates as above and oligonucleotides IF08 and IF09. The PCR products were digested with BamHI and HindIII and inserted into the same sites of pKS84 [15]. In order to construct similar fusions to mCherry, a derivative of pKS84 was engineered in which the *URA3* marker was substituted by *LEU2* and GFP replaced by mCherry. The exchange of the marker was made by PCR amplification from pACT2-1 (Clontech) with oligos IF37 and IF38, digestion of the product with SalI and XmaI and subcloning in the same sites of pKS84. Insertion of mCherry was then carried out by subcloning a PCR product obtained by amplification from pXDC50 with oligos IF39 and IF40 and digestion with HindIII and SalI, leading to a *Pgal-mCherry* fusion in plasmid pIF215. Two DNA fragments containing *vipA* or *vipA-1* were removed from pIF206 or pIF209 (see above) by digestion with BamHI and HindIII and inserted in the same sites of pIF215, yielding pIF216 and pIF217 carrying the *vipA-mCherry* fusions.

For expression of his-tagged proteins in *E. coli*, wild-type or mutant vipA-1 alleles were amplified using primers NSP22 and NSP23, containing NdeI and BamHI sites. The PCR products were digested with these enzymes and inserted in the same sites of pET15b (Novagen), and plasmids pET15b-VipA and pET15b-VipA-1 were obtained. Plasmid pNSvipA was constructed by amplifying *vipA* with oligos NSP29 and NSP30, both containing SalI sites. The PCR product was digested with this enzyme and cloned in the SalI site of pNS00 [13].

### *vipA* linker mutagenesis

Mutagenesis was performed using the GPS Linker Scanning System according to the manufacturer's instructions (New England Biolabs) and plasmid pNSvipA used as a template (see above). The transposition reaction was performed with 1.5 µg pNSvipA incubated with 20 ng pGPS4 and 1 µl TnsABC. Dilutions of the reaction were used to transform ElectroTen Blue *E. coli* (Stratagene) by electroporation and plated on LB+Carbenicillin+Chloramphenicol. Plasmid DNA was prepared from approximately 25000 scraped colonies, transformed into the yeast strain NSY01 and transformants plated on SC-ura/fructose. Invertase overlay was performed on yeast transformants as previously described [13] and roughly 14% of colonies were white, indicating a restoration of the Vps^+^ phenotype. 100 of these colonies were isolated, grown overnight in liquid SC-ura/fructose and plasmid DNA extracted. Plasmid pools were digested with PmeI to excise the Cm^R^ marker, the plasmid backbone gel purified, self-ligated and transformed into *E. coli*. Plasmid DNA was extracted from 352 colonies and transformed into NSY01. Roughly 30% of the colonies were white on the Invertase Overlay, from which 40 were grown overnight in liquid SC-ura/fructose, plasmid DNA prepared and sequenced to map and identify the linker insertion.

### Immunodetection of VipA and GFP fusions

For detection of VipA in *L. pneumophila*, strains were grown overnight in AYE medium with the appropriate antibiotics. Bacteria (approximately 2.8×10^8^ cells) were harvested by centrifugation, sample buffer was added and after SDS-PAGE the proteins were immunoblotted with affinity-purified rabbit polyclonal antibody raised against recombinant His_6_-VipA (essentially as described by Zhu *et al.* [17]). For detection of GFP or GFP fusions in yeast, strains were grown on SC-ura+Fructose or +Galactose plates for three days at 30°C and several colonies were picked and grown overnight in identical same liquid medium. An amount equivalent to OD_600_ 3 in a total volume of 40 µl of SDS-Loading Buffer (or OD_600_ 1 in 15 µl in the case of the strain expressing GFP) was boiled for 5 minutes and run on SDS-PAGE. Transfer and immunoblotting were performed as above using a rabbit polyclonal antibody to GFP at a 1∶1000 dilution. For immunoblots of translocated VipA, differentiated THP-1 cells were infected with *L. penumophila* strains for 3 hr at an MOI = 50. Cells were lysed by incubation with PBS+Triton X 2% for 15 min at 4°C. Lysates were centrifuged at 13.000 rpm for 15 min, and pellet resuspended in the same solution.

### Overexpression and purification of His_6_-VipA and His_6_-VipA-1

*E. coli* BL21(DE3) strains carrying *vipA* expressing plasmids (see Table S2) were grown overnight and backdiluted 1∶50. At an OD_600_∼0.6, IPTG (isopropyl β-D-1-thiogalactopyranoside) was added to a final concentration of 1 mM and growth was continued for 2 hours. For pull-down assays, pellets were resuspended in 20 mM Tris-HCl pH 8.0, 300 mM NaCl, 1 mM EDTA, 100 µg.ml^−1^ PMSF+Protease Inhibitor Cocktail (Sigma), 1 mg.ml^−1^ lysozyme and incubated for 30 min on ice. Bacteria were lysed using 3 passages in a French Press, lysates were centrifuged at 16000 rpm and supernatants were transferred to tubes containing Ni-NTA beads (QIAgen) or loaded on HisTrap FF Columns (GE Healthcare) connected to a AKTA FPLC system (GE Healthcare), and eluted with Imidazole gradients.

### Tissue culture and growth of cell lines

*Acanthamoeba castellanii* were cultured in PYG medium at 28°C without agitation. CHO FcγRII and THP-1 cells were grown in DMEM or RPMI, respectively, supplemented with 2 mM glutamine and 10% heat-inactivated fetal bovine serum, at 37°C in a 5% CO_2_ incubator. Differentiation of monocytes was accomplished 3 days after the addition of 1 ng.ml^−1^ of PMA (phorbol 12-myristate 13-acetate) to the medium.

### Mammalian cell immunofluorescence and confocal microscopy

CHO-FcγRII cells [18] were grown, transfected, fixed and permeabilized for immunofluorescence as described previously [15]. Actin or DNA staining was carried out by incubating cells with, respectively, Rhodamine-phalloidin (Sigma, 5 µg.ml^−1^) or DAPI (100 µg.ml^−1^) during 30 min. Infection of THP-1 monocyte-like cells was carried out as described above using an Multiplicity of Infection of 50, and cells processed for immunofluorescence (14). For rhodamine-dextran endocytosis assays, the compound was added at 4 hr post-infection, for 1 hr and chased for 10, 30, 120 and 240 min, at 1 mg.ml-1. Mouse monoclonal primary antibodies used were α-EEA1 (BD Biosciences), α-LAMP1 (UH1), α-ALIX (Biolegend), and αKDEL (Santa Cruz Biotecnology). Secondary antibodies were goat anti-mouse Alexa-594 or FITC-labeled (Invitrogen), or α-rabbit Alexa-647 (Invitrogen), -TRITC or -FITC (Sigma). The αVipA antibody used in immunofluorescence assays was previously affinity-purified (see above). Microscopy was carried out on Laser Scanner Confocal Microscopes (Zeiss LSM710, Leica SP5 II STED-CW or Leica TCS SP2).

### Colocalization quantification and statistical analysis

Quantitative analysis of colocalization was performed by calculating the Manders overlap coefficient, corresponding to the fraction of green pixels (VipA-EGFP signal) that overlap with red pixels in relation to the total green pixels [19]. For this purpose, signal intensities for each cell (n>15 for each antibody) were adjusted in ImageJ and the coefficients determined with the plugin JACoP [20]. Statistical significance was determined with unpaired t test, and p values obtained are indicated (**, p<0.01; ***, p<0.001).

### Yeast microscopy and FM4-64 staining

*S. cerevisiae* cells expressing GFP or mCherry fusion proteins were grown in plates with SC-Ura/-Leu/-Ura-Leu supplemented with 2% fructose at 30°C for 3 days. Several colonies were inoculated in identical liquid medium supplemented with 2% galactose and grown overnight. The next day the cultures were diluted in the same medium to an OD_600_ = 0.3, and grown to an OD_600_ = 0.5. FM4-64 staining was performed essentially as previously described [15]. Cells were mounted on agarose pads and visualized on a Laser Scanner Confocal Microscope (Zeiss LSM710).

### Pull-down assays

For pull-down assays, U937 monocyte post-nuclear supernatants (PNS) were prepared by harvesting 2×10^8^ U937 cells grown in suspension in RPMI+Glu+10%FBS. Cells were lysed by addition of 10 ml 50 mM Tris-Hcl pH 8, 150 mM NaCl, 0.1 mM EDTA, 0.5% NP-40, 1 mM DTT, 0.1 mM NaVO_4_, 100 µg.ml^−1^ PMSF+Protease Inhibitor Cocktail (Sigma). Lysis was accomplished by 1 hour incubation on ice and centrifugation at 4000 rpm for 30 min. 10 ml U937 PNS were mixed with 100 µl Ni-NTA beads loaded with approximately 100 µg his-tagged bait proteins, incubated at 4°C and washed with His buffer (20 mM Tris-HCl pH 8.0, 300 mM NaCl, 10% Glycerol, 100 µg.ml^−1^ PMSF)+40 mM Imidazole. Beads were applied to a BioRad column, washed again and eluted with HisBuffer+500 mM Imidazole. Eluates were collected, mixed with Protein Sample Buffer and analyzed by SDS-PAGE followed by either Coomassie staining or Western blot. Differential protein bands in eluates that were absent in His-FabI and Beads only pull-downs, and did not react with antibody against polyHistidine were excised, digested with trypsin and analyzed by Liquid Chromatography/Mass Spectrometry (LC/MS). For Western blotting 1∶5000 dilutions of monoclonal antibody against either actin or polyHistidine (Sigma) were used.

### Actin polymerization assays

G-actin and Pyrene-labeled G-actin (Cytoskeleton, >99% pure) were prepared as indicated by the manufacturer and kept in G-actin buffer (Tris-Cl 5 mM, CaCl_2_ 0.2 mM, ATP 0.2 mM, DTT 1 mM). Before polymerization, conversion to Mg-actin was accomplished by addition of EGTA to 0.2 mM and MgCl_2_ to 50 µM and incubation for 2 min at room temperature. Polymerization assays were made on black bottom microplates and fluorescence read in a microplate reader equipped with an injector (Infinite M200, Tecan). Values were obtained using an excitation wavelength of 365 nm and emission of 407 nm, and recorded at 10 sec intervals. Data were collected with Magellan software v6.4 (Tecan) and then processed in Excel (Microsoft). Reactions contained 2 µM actin (10% Pyrene-labeled), and were initiated by adding KMEI Polymerization Buffer (Imidazole 10 mM pH 7, KCl 50 mM, MgCl_2_ 1 mM, EGTA 1 mM). Purified His_6_-VipA or His_6_-VipA-1 in G-Mg buffer (G-actin buffer with 0.1 mM MgCl_2_ instead of Cacl_2_) were added as indicated.

To measure elongation, actin seeds were prepared as follows (adapted from [21]). Actin was resuspended in G-actin buffer to a final concentration of 15 µM, converted to Mg-actin for 20 min and polymerization induced by addition of KMEI Polymerization buffer (see above) and incubation for 2 hr at room temperature. To stabilize filaments, actin was diluted to 5 µM in the presence of 5 µM of phalloidin for 5 min. The polymerized actin was recovered after centrifugation for 20 min at 90,000 rpm and 4°C in a TLA100 rotor and these protected filaments ressuspended in G-actin buffer and used in elongation assays with 1 µM G-actin. Filament concentration and rate of monomer addition were calculated as described previously [22].

### Accession numbers

Swissprot IDs of proteins: *Legionella pneumophila* VipA, Q5C8M7; *Chlamydia trachomatis* TARP, Q6GX35; *Salmonella enterica* serovar *typhimurium* SipC, Q56020, and SipA, P0CL52; *Vibrio parahaemolyticus* VopL, B9A807, and *Vibrio cholerae* VopF, B5AN40; *Rickettsia conorii* Sca2, Q92JF7; *Saccharomyces cerevisiae* Bro1, P48582; *Homo sapiens* Alix, Q8WUM4.

## Results

### *L. pneumophila* effector VipA binds actin *in vitro*

**Figure 1:**
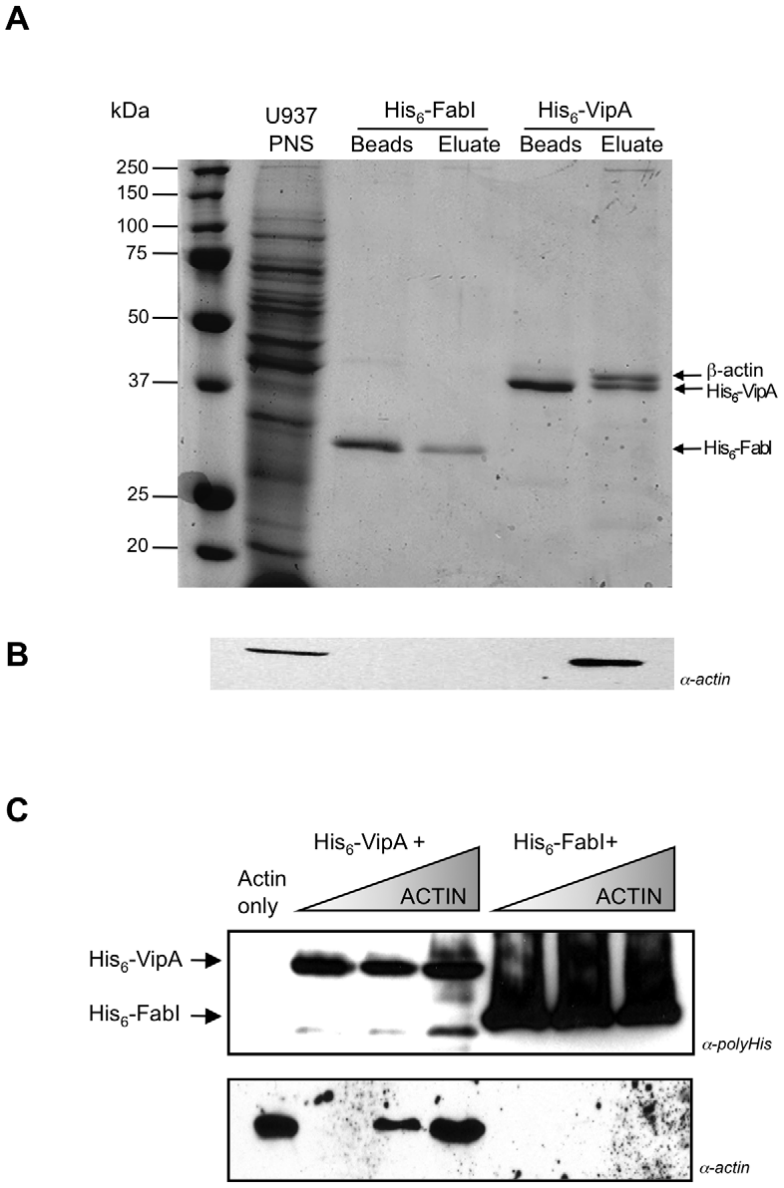
*In vitro* interaction analysis between His_6_-VipA and actin by pull-down assays. **A.** U937 cell Post-Nuclear Supernatants (U937 PNS) were incubated with Ni-NTA agarose beads preloaded with His_6_-VipA or His_6_-FabI (see Materials and Methods for details). After washing, bound proteins were eluted with 500 mM Imidazole. Samples containing preloaded beads or eluates were resolved by SDS-PAGE and bands visualized by Coomassie staining. The differential band appearing in the VipA eluate (arrow) was excised and identified by LC/MS as β-actin. **B.** Similarly prepared samples were resolved by SDS-PAGE, transferred to nitrocellulose and immunoblotted with an anti-actin monoclonal antibody (α-actin). **C.** Ni-NTA agarose beads preloaded with His_6_-FabI or His_6_-VipA were incubated with increasing concentrations of purified G-actin, washed and eluted. Eluates were resolved by SDS-PAGE, transferred to nitrocellulose and Western-blotted with an anti-histidine antibody (α-polyHis, panel above) or an anti-actin monoclonal antibody (α-actin, panel below).

In previous work aimed at identifying new effectors [13], disruption of organelle trafficking was observed when VipA was ectopically expressed in yeast, raising the possibility that an interaction between VipA and host cell proteins could be involved in the process. We sought to identify eukaryotic binding partners for VipA in macrophages by *in vitro* assays using purified VipA as bait to pull-down proteins from U937 human monocyte-like cell extracts. For this purpose, a recombinant version of VipA carrying a 6-histidine tag at its N-terminal region (His_6_-VipA) was constructed in plasmid pET15b (Novagen) and purified by Ni-NTA affinity chromatography. A post-nuclear supernatant was prepared from U937 cells and incubated with Ni-NTA agarose beads, preloaded either with His_6_-VipA or an irrelevant protein, His_6_-FabI, the *L. pneumophila* enoyl acyl CoA reductase protein, used as negative control for the interaction as it is not translocated into the host cell [23]. Bound proteins were eluted with 500 mM imidazole, separated by SDS-PAGE and analyzed by Coomassie staining (Figure 1A). A 42-kDa band that co-eluted with VipA but was absent in the FabI eluate and did not react with an antibody against polyHistidine (data not shown) was excised and identified as β-actin by Liquid Chromatography/Mass Spectrometry (LC/MS). This result was confirmed with a Western-blot using a monoclonal antibody against actin (Figure 1B). To test if the interaction between VipA and actin was direct, additional pull-down assays were performed using His_6_-VipA and increasing concentrations of purified monomeric G-actin (Figure 1C). The results show that actin bound to VipA directly, without the requirement of any additional host factor.

### Interference of VipA in trafficking pathways is linked to actin binding

**Figure 2:**
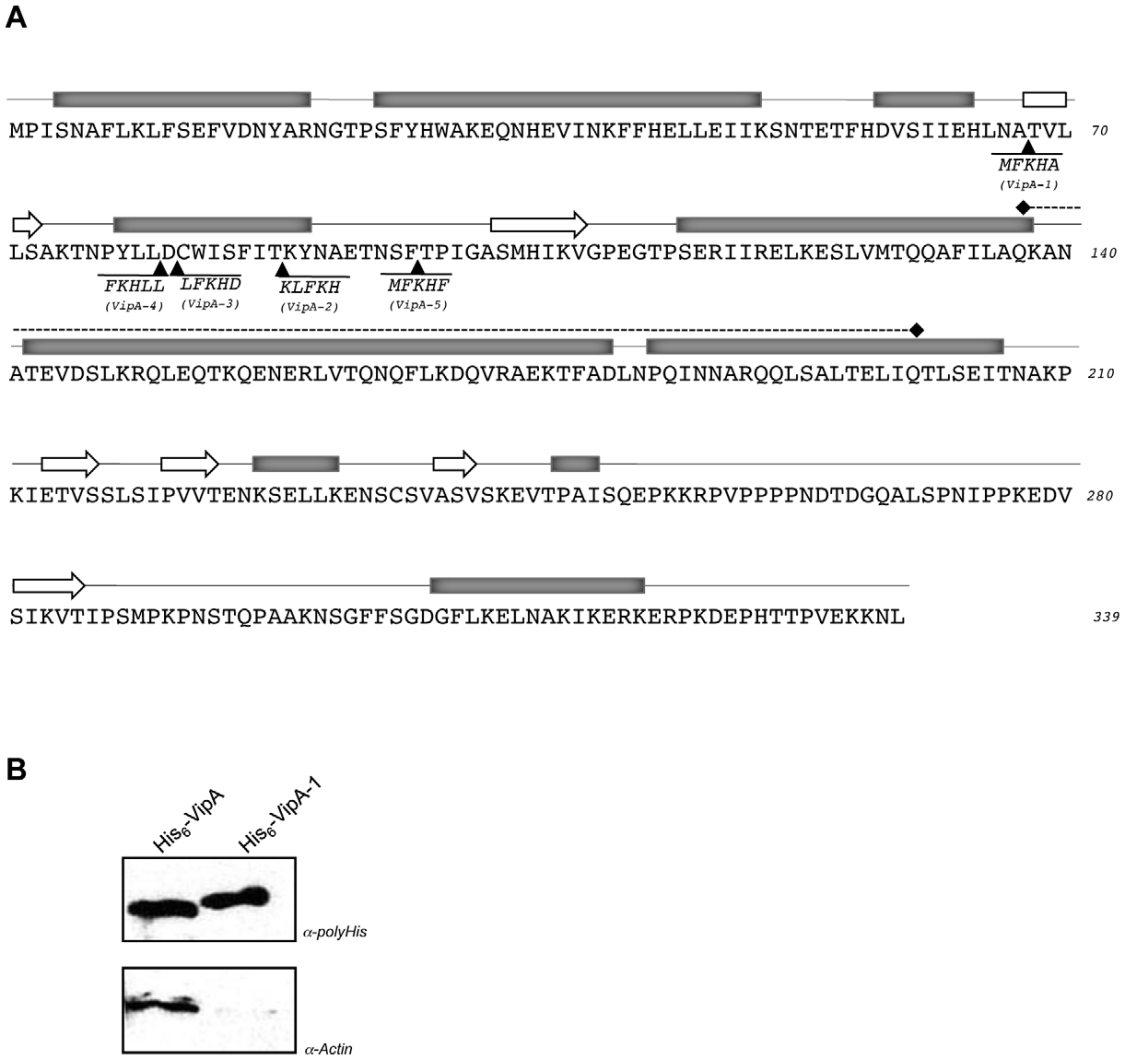
Vps ^+^ phenotype of VipA mutants is linked to actin binding. **A.** Primary sequence of VipA and localization of in-frame insertions in mutant proteins encoded by the 5 obtained Vps^+^ alleles (*vipA-1* to *vipA-5*). The inserted residues in each allele are shown below the sequence. The predicted secondary structure is depicted, with alpha-helices as rectangles, beta-strands as block arrows (PSIPRED v2.6 software [54] and the central coiled-coil motif as dashed line (SMART software [55], [56] **B.** Pull-down assays with G-actin and wild-type VipA or mutant VipA-1 proteins. U937 Post-nuclear supernatants were incubated with Ni-NTA agarose beads preloaded with His_6_-VipA or His_6_-VipA-1, washed and eluted with 500 mM Imidazole. Eluates were resolved by SDS-PAGE, transferred to nitrocellulose and analyzed by Western-blot with monoclonal antibodies against polyHistidine (α-polyHis) or against actin (α-Actin).

The finding that VipA interacted directly with actin *in vitro* suggested a mechanistic basis for the ability of the protein to affect vesicle trafficking pathways in yeast, as actin is involved in numerous membrane trafficking processes [24]. To test if the ability of VipA to disrupt trafficking depended on its interaction with actin, we generated linker mutant alleles of *vipA* using the transposon-based GPS system (New England Biolabs). This procedure ultimately introduces 15-bp insertions in the target gene, of which 1/3 result in the insertion of a stop codon and 2/3 in the in-frame insertion of five amino acids. A plasmid harboring *vipA* was mutagenized and the library of mutants (approximately 25000 colonies) transformed into yeast strain NSY01, used as a reporter for screening Vacuolar Protein Sorting defects (Vps^−^ phenotype) [13]. This strain produces the hybrid protein Carboxypeptidase Y-Invertase, which travels to the vacuole in wild-type yeast but can be missorted to the cell surface if vacuolar protein trafficking is disrupted, which is the case when wild-type VipA is ectopically expressed. The excreted enzyme hydrolyses sucrose present in the medium that can be detected by the brown color of the colonies in a particular screening medium (Vps^−^ phenotype; see [25]). In this work, we were interested in isolating *vipA* mutants that no longer disrupted vacuolar traffic, thus leading to the formation of white/Vps^+^ colonies. These colonies were isolated from transformants of the *vipA* linker library, and the corresponding plasmids sequenced to map the insertions. Five different in-frame insertions were identified disrupting the ability of the protein to cause a Vps^−^ phenotype (Figure 2A). All five VipA mutant proteins were stably expressed in yeast at levels identical to wild-type (data not shown). Interestingly, all insertions mapped between codons 67 and 98, just upstream of a sequence encoding a predicted coiled-coil domain located between residues 137 and 200. Sequence analysis of this region and additional investigation of the entire protein sequence did not reveal further homologies to known domains or motifs, although we noted a high occurrence of prolines in the C-terminal region of VipA.

To further understand the importance of the N-terminal region, one of these mutant proteins (VipA-1, insertion at residue 67) was expressed and purified as a his-tagged fusion protein (His_6_-VipA-1), displaying expression and solubility levels similar to wild-type VipA. Its ability to bind actin was tested as above in pull-down assays using U937 cell extracts, where a significant decrease in affinity for monomeric actin was observed (Figure 2B). Taken together, these results show that the ability of VipA to disrupt vacuolar trafficking in yeast and its ability to bind actin are linked. This result is inconsistent with a non-specific association of actin and VipA.

### Direct enhancement of actin polymerization by VipA *in vitro*

**Figure 3:**
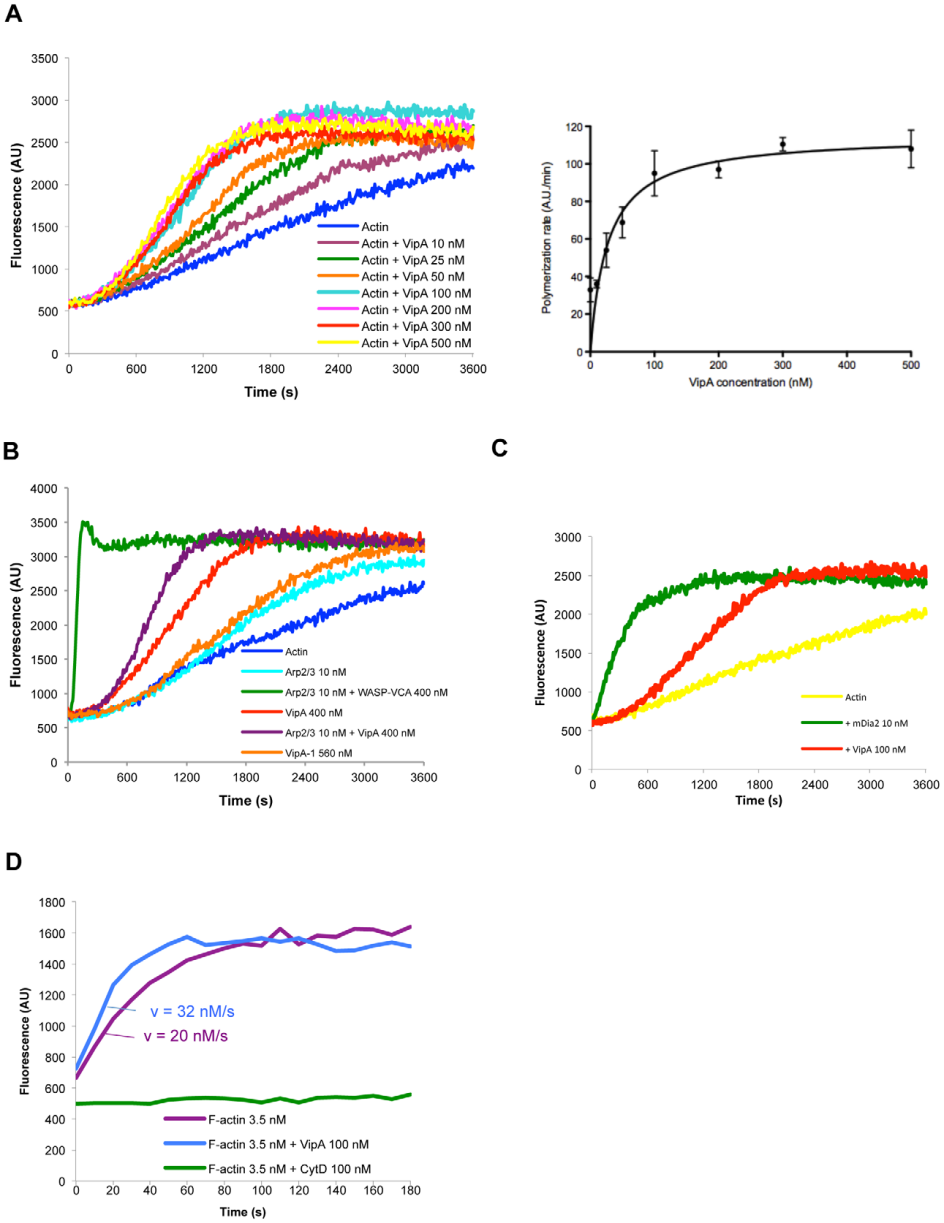
*In vitro* actin polymerization assays. Samples contained 2 µM monomeric actin (10% Pyrene-actin) and fluorescence (expressed in Arbitrary Units, AU) was measured over time after initiation of polymerization. **A.** The displayed concentrations of His_6_-VipA were added to the samples and polymerization initiated. The graph on the right is a plot of the average and standard deviation for the rates of actin polymerization for each His_6_-VipA concentration. Velocities were determined for the interval 600–1200 s using the results of two independent experiments. **B** and **C.** Actin samples contained 10 nM Arp2/3 complex, 400 nM WASP-VCA (Cytoskeleton), 400 nM His_6_-VipA, 560 nM His_6_-VipA-1 and 10 nM of mDia2 where indicated. **D.** Elongation of filaments was carried out using phalloidin-stabilized actin seeds and 1 µM of G-actin (10% Py-labeled), in the presence of His_6_-VipA-1 (100 nM) or Cytochalasin D (100 nM). Determination of F-actin concentration and rates of polymerization are described in Materials and Methods.

To find out whether VipA had any effect on the assembly of actin filaments we used pyrene-actin polymerization assays. Fluorescence of pyrene-actin increases significantly when G-actin monomers are incorporated into a filament, permitting polymerization to be measured in real-time. In reactions containing 2 µM actin (10% pyrene-labeled), His_6_-VipA stimulated actin polymerization at nanomolar concentrations in a dose-dependent manner (Figure 3A). Saturation occurred at approximately 100 nM VipA, a concentration that increased the actin polymerization rate by approximately 3-fold (Figure 3A, right panel). This effect on microfilament polymerization is not as potent as the nucleation of actin by the Arp2/3 complex activated by WASP-VCA (Figure 3B), or the mouse formin mDia2 (Figure 3C). Also, in contrast to the case of other bacterial effectors, His_6_-VipA does not activate Arp2/3-mediated actin polymerization, as the effect seen in the presence of inactive Arp2/3 and VipA is additive of the two individual effects. The VipA-1 mutant displayed only a small effect on the assembly of actin filaments, in the same order of magnitude of inactive Arp2/3, even at high concentrations (Figure 3B).

Polymerization of actin occurs in two phases with distinct kinetics. The initial and limiting step, nucleation, occurs very slowly and leads to the formation of actin trimers, whereas the subsequent elongation of the filament takes place at a much higher rate. The observed shorter duration of the initial lag phase in the presence of VipA was consistent with a role in enhancing actin nucleation. To find out if VipA was also affecting elongation, we carried out polymerization assays with actin seeds. In these assays all polymerization occurs from short preformed actin filaments, and thus increases in fluorescence are not due to *de novo* actin nucleation. As shown in Figure 3C VipA enhanced slightly but reproducibly the elongation rate of the small filaments. Cytochalasin D, a well-characterized filament barbed-end capper used here as a negative control, lead to a complete halt in elongation. The effect mediated by VipA during filament elongation could not, however, account for the overall increase in actin polymerization observed above (Figures 3A and B), which indicates VipA is acting predominantly as a nucleator.

Taken together, the data obtained with the *in vitro* actin polymerization assays shows that VipA is able to enhance actin polymerization without the requirement of additional proteins. Moreover, these results indicate the effector uses a mechanism that favors nucleation and moderately increases the rate of addition of monomers during elongation.

### VipA is not essential for entry or replication in *A. castellanii* and macrophages

The *L. pneumophila* genome encodes approximately 300 effector proteins that are translocated to the host cell during infection. Surprisingly the vast majority of effector genes are dispensable for intracellular replication and yield no obvious phenotype when deleted. In order to determine if VipA is essential for infection of host cells, the *vipA* gene was deleted from Legionella strain KS79. The wild-type *vipA* allele was substituted by a kanamycin-resistance cassette and the absence of VipA protein in the resulting strain LPIF3 was confirmed by Western immunoblot using a polyclonal anti-VipA antibody (Figure S1A). Infection of THP-1 macrophages and the amoeba *A. castellanii* was carried out with Legionella strains KS79 and LPIF3 harboring plasmid pXDC31 (Table S2), in which GFP expression is driven by the IPTG-inducible *Ptac* promoter. Thus, intracellular bacterial replication can be followed by real time monitoring of GFP fluorescence measurements [26]. In addition, the translocation-defective *dotA* mutant strain containing the same plasmid was used as a negative control for intracellular growth. Replication in *A. castellanii* was not affected by deletion of *vipA*, and identical results were obtained using THP-1 macrophages as hosts (Figure S1B; data not shown). As a more subtle effect in the initial phase of phagocytosis may be difficult to detect in this assay, gentamicin protection assays were carried out to measure entry into host cells. Similarly, no significant difference was observed between the number of intracellular bacteria from wild-type or *vipA* strains (data not shown).

### VipA forms puncta in yeast that localize to actin-rich regions

**Figure 4:**
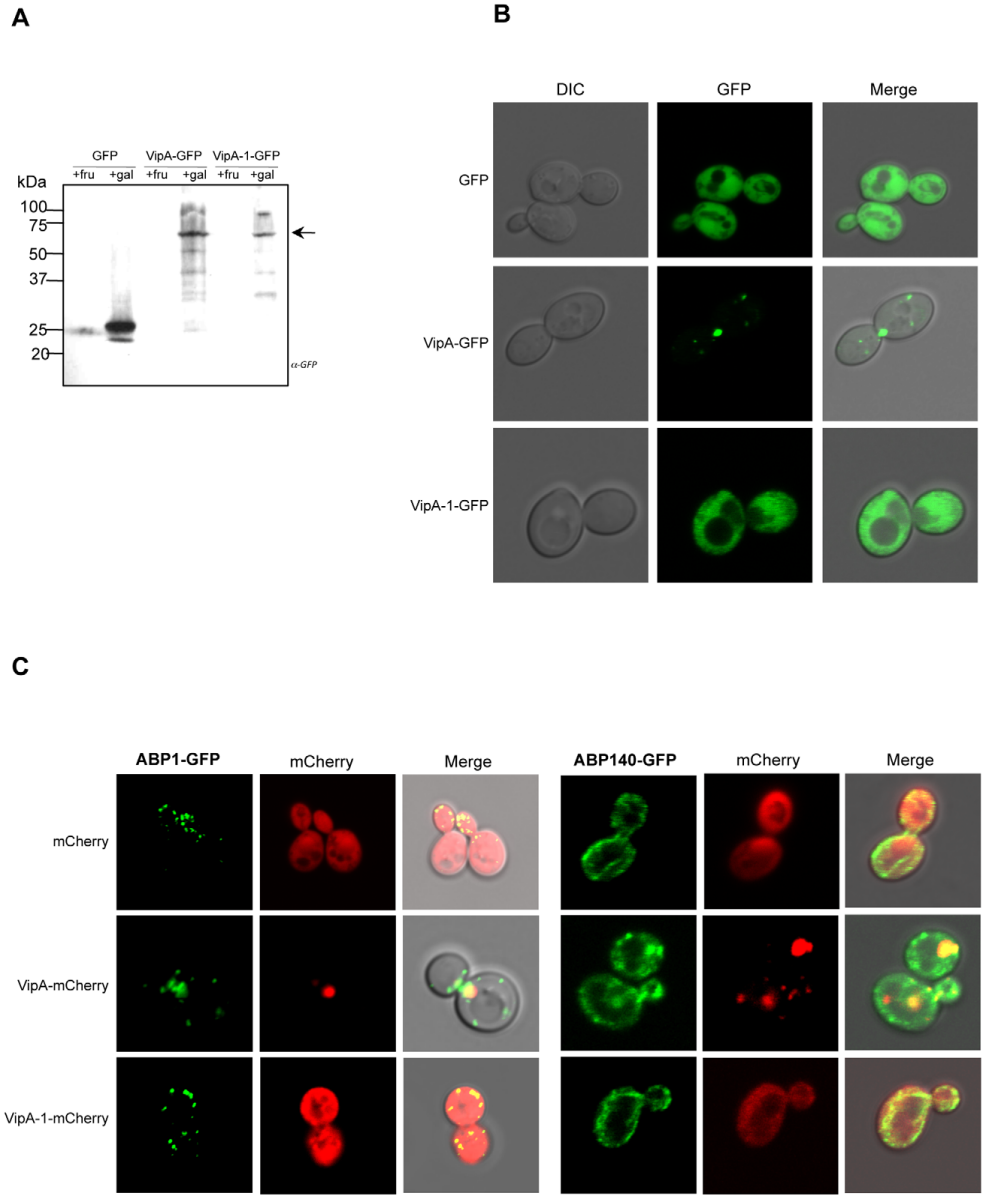
Localization of VipA-GFP in *S. cerevisiae*. **A.** Yeast strains producing GFP, VipA-GFP or VipA-1-GFP (respectively strains SCIF00, SCIF01 and SCIF02) were grown in medium with fructose or galactose to an OD_600_∼0.5. Equivalent amounts of cell lysates were run on SDS-PAGE and analysed by Western-blot using an anti-GFP polyclonal antibody [57]. The arrow points to the bands corresponding to VipA-GFP or VipA-1-GFP fusion proteins. **B.** The same strains grown in galactose were viewed using Laser Scanning Confocal microscopy and representative images are shown (left). **C.** Colocalization of VipA-mCherry and actin structures in yeast. Yeast strains producing mCherry, VipA-mCherry or VipA-1-mCherry and Abp1-GFP (left) or Abp140-GFP (right; see Table S1) were grown and visualized as above.

To gain further insight into the function of VipA we analysed its subcellular localization in eukaryotic cells. For this purpose initial studies were performed in *S. cerevisiae*, in which the Vps^−^ phenotype had led to *vipA* identification. *S. cerevisiae* strain NSY01 (see above) was transformed with plasmids carrying C-terminal GFP fusions of either wild-type VipA or mutant VipA-1 under the control of the galactose-inducible *Pgal* promoter. In the resulting strains, expression of the ∼70 kDa recombinant proteins in the presence of the inducing sugar was confirmed by Western-blot of the cell extracts using an anti-GFP antibody (Figure 4A). Laser Scanning Confocal Microscopy analysis revealed the localization of VipA-GFP in puncta in yeast cells (Figure 4B), whereas the VipA-1-GFP mutant protein exhibited homogeneous cytosolic distribution, similar to GFP alone. Interestingly, approximately half of the VipA puncta were associated with the mother-bud neck in dividing cells, a site containing the cytokinetic ring and enriched in actin filaments, which is consistent with the ability of VipA to interact with actin *in vitro* (see above). Thus, the association of VipA with other actin-rich structures was investigated. In *S. cerevisiae*, visible F-actin structures consist of patches, cables and rings. Patches are highly motile punctate structures that form at the cell cortex, mediate endocytosis and contain numerous actin-associated proteins. After endosome internalization they transition to a phase of rapid movement that depends on their transport along actin cables away from the cell membrane [27]–[29]. Actin cables consist of bundles of F-actin aligned along the mother-bud axis and serve as tracks for the movement of secretory vesicles, mitochondria, Golgi, and vacuoles from the mother cell to the growing bud [30], [31]. Association of VipA-mCherry with actin in patches and cables was assessed by colocalization studies with two actin markers: Abp1-GFP, which binds F-actin patches in endosomal sites, and Abp140-GFP, which associates with microfilaments in both patches and cables [27], [32], [33]. The distribution of VipA-mCherry and VipA-1-mCherry was identical to the observed above with the VipA-GFP and VipA-1-GFP fusions, respectively (Figure 4C). Colocalization between VipA and actin structures was observed in the case of Abp1-containing endosomes (Figure 4C, left panel) and for Abp140-associated patches, but not cables (Figure 4C, right panel). The presence and distribution of these markers was not affected by the presence of either VipA or VipA-1-mCherry. These results show that the puncta formed by VipA in yeast are located in sites containing a high array of actin filaments, namely the cytokinetic ring and cortical patches, and that the *vipA*-1 mutation that decreased the affinity of VipA for actin *in vitro* also abolished its targeting to these locations.

### VipA associates with components of the yeast MVB pathway

**Figure 5 ppat-1002546-g005:**
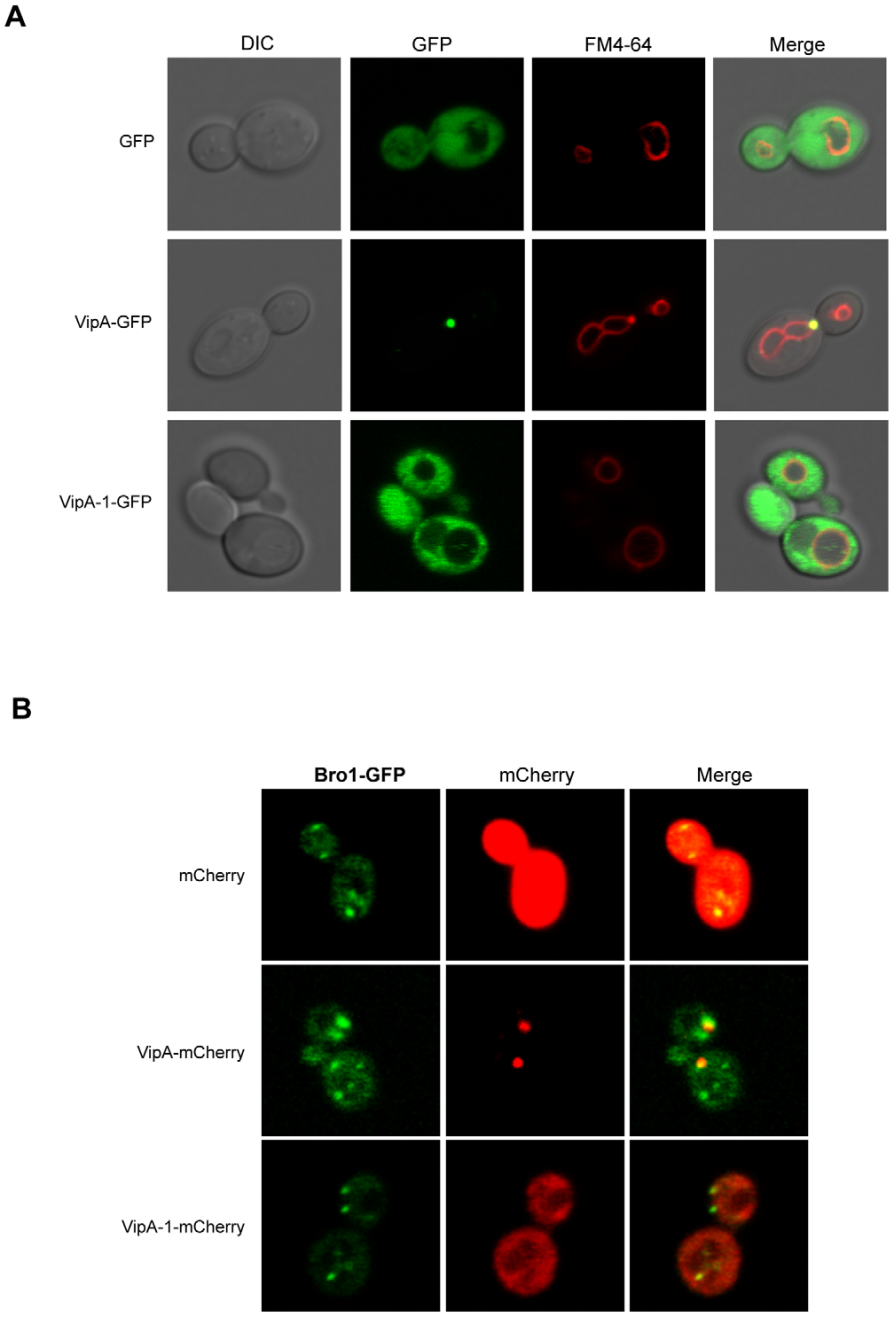
VipA associates with components of the yeast MVB pathway. **A.** For vacuole staining, *S. cerevisiae* strains were grown as above (see legend of Figure 4), harvested and additionally pulse-chased with N-(3-triethylammoniumpropyl)-4-(p-diethylaminophenylhexatrienyl)-pyridinium dibromide (FM4-64). **B.** Strains carrying the fusions Bro1-GFP (left) and mCherry, VipA-mCherry or VipA-1-mCherry were grown and visualized as above.

The interference of VipA in the formation of the MVB in yeast [13] strongly hinted its interaction with one or more components of this pathway. MVBs are present in both the Endocytic and Secretory/Biosynthetic pathways and result from the invagination and budding of the endosome membrane, and eventually fuse and deliver their cargo to the vacuole/lysosome. We initially tested an interaction with the yeast vacuole using the styryl dye FM4-64, which stains the vacuole membrane after being internalized by endocytosis [34]. Staining of cells producing VipA-GFP revealed that approximately 70% of the VipA puncta were associated with the vacuole membrane and co-localized with an abnormal pre-vacuolar compartment observed previously in strains expressing VipA (Figure 5A; [13]). This structure is identical to the one characteristic of class E *vps* mutants, where defective MVB formation leads to the inability of endocytosed cargo to fuse completely with the vacuole [35], [36]. This failure is caused mainly by defects in packaging of the cargo into intralumenal vesicles of MVBs, which is carried out by the sequential action of proteins composing the ESCRT 0-III complexes (Endosomal Sorting Complex Required for Transport). One of the proteins involved in these late steps of the MVB pathway is Bro1, a cytoplasmic yeast protein that transiently associates with endosomes, where it is required for the formation of intralumenal vesicles. We assessed VipA-mCherry colocalization with Bro1-GFP and verified that approximately 37% of VipA puncta colocalized with Bro1-GFP (Figure 5B), confirming an association of the effector with this protein component of the MVB.

Taken together, these results show that VipA associates with components of the yeast MVB pathway, namely Bro1-containing endosomes and the vacuole. Moreover, they suggest that the previously observed effect of VipA on mistrafficking of vacuolar proteins could be due to a defect on late steps on the MVB pathway wherein fusion of endosomes with the vacuole occurs.

### VipA colocalizes with host cell actin and early endosomes but not the LCV during macrophage infection

**Figure 6 ppat-1002546-g006:**
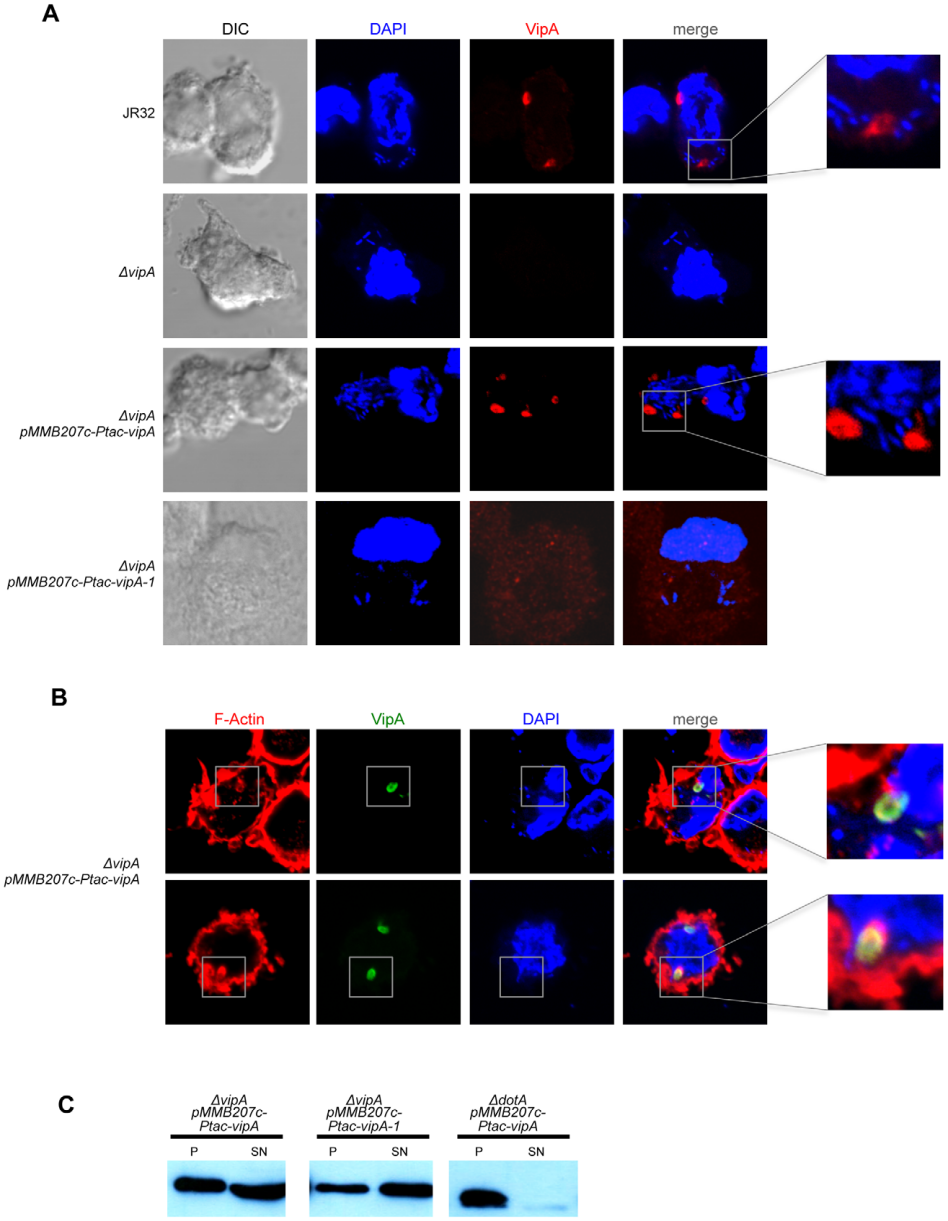
Localization of translocated VipA and VipA-1 during infection of THP-1 macrophages. THP-1 macrophages were infected with *L. pneumophila* strains (JR32, *ΔvipA*, *ΔvipA pMMB207c-Ptac-vipA* or *ΔvipA pMMB207c-Ptac-vipA-1*) with an MOI = 50 for 8 hours. Immunofluorescence was carried out as described (Materials and Methods). DNA was stained with DAPI and VipA with a purified polyclonal anti-VipA antibody. **A.** and F-actin was stained with Rhodamine-phalloidin. **B.** Representative confocal microscopy images are shown. **C.** Immunoblot of samples containing lysates of THP-1 macrophages infected for 3 hr with the indicated *L. pneumophila* strains at an MOI = 50 (see Material and Methods). P, lysate pellet; SN, lysate supernatant.

**Figure 7:**
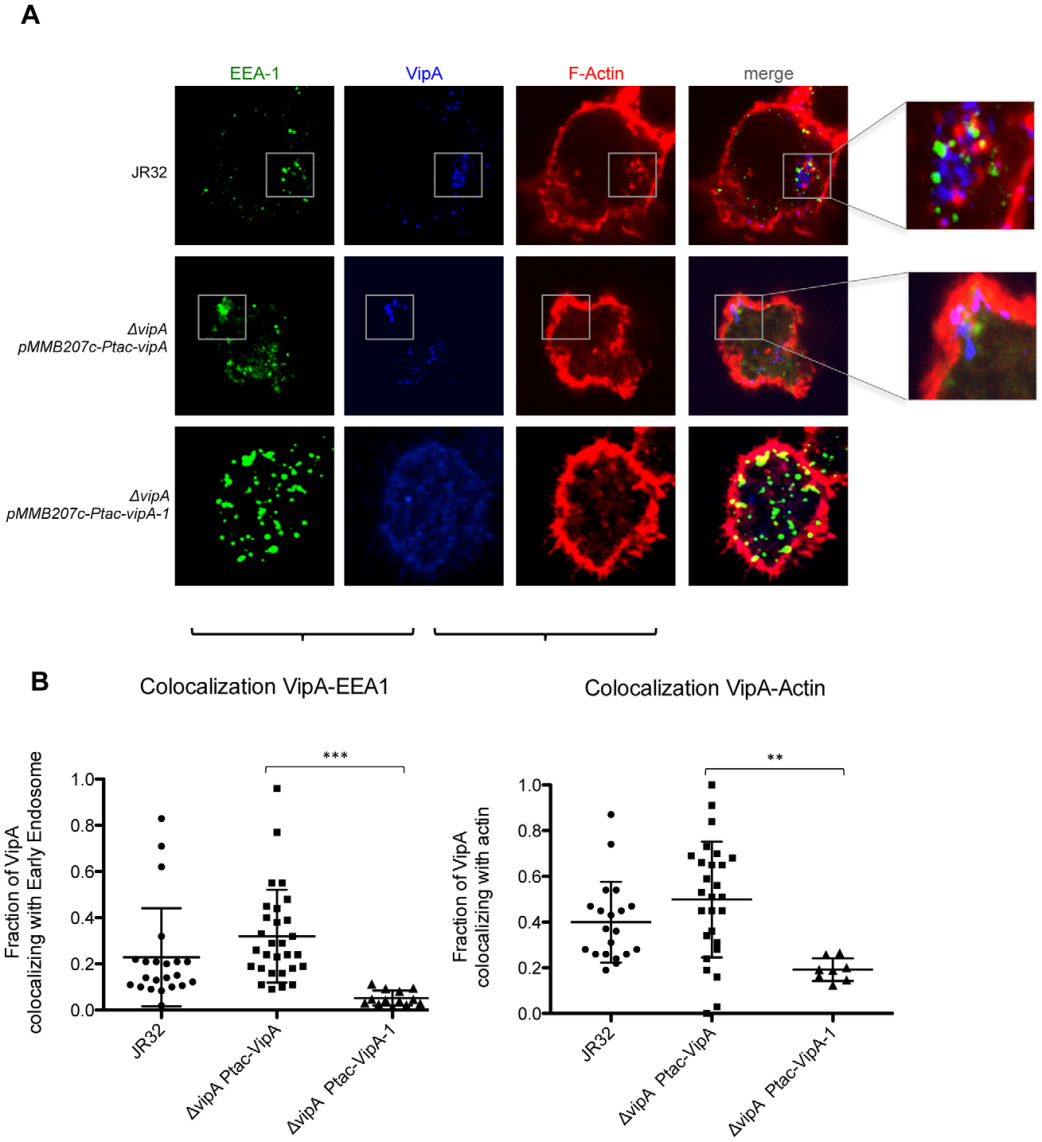
Colocalization of VipA with host cell early endosomes and actin in macrophage infection. **A.** THP-1 macrophages were infected as above (see Legend of Figure 6 and Materials and Methods). F-Actin was stained with Rhodamine-phalloidin and Early Endosomes using an αEEA-1 antibody. **B.** Quantification of colocalization between VipA and F-Actin or EEA-1 was performed by calculating the Manders coefficient using ImageJ and the Plugin JACoP (see Materials and Methods for details). The Manders coefficient corresponds to the fraction of VipA-GFP or VipA-1-GFP (blue signal) overlapping with each marker (green or red signal) divided by the total green signal in an image. Filled symbols represent cells expressing VipA-GFP (n>15), open symbols cells expressing VipA-1-GFP, and bars indicate mean and standard deviation. Statistical analysis was performed with unpaired t test, and p values obtained are indicated (**, p<0.01; ***, p<0.001).

In order to assess the subcellular localization of VipA in host cells under physiological conditions, infection of THP-1 monocyte-like cells was carried out, cells fixed at several time points post-infection, processed for immunofluorescence using a polyclonal anti-VipA antibody and analysed using Laser Scanning Confocal Microscopy. After infection with wild-type strain *L. pneumophila* JR32, VipA was found in diverse structures inside the host cell, which varied in size from puncta to larger formations (Figures 6 and 7). VipA was not associated with the LCV at any time point from 30 min to 14 hr after uptake (Figure 6A and B; data not shown). To assess the localization of the VipA-1 mutant, *L. pneumophila vipA* null-mutant background strains were constructed carrying plasmids encoding IPTG-inducible copies of either the wild-type or the mutant *vipA* allele (respectively, *ΔvipA* pMMB207c-*Ptac-vipA^+^* or *ΔvipA* pMMB207c-*Ptac-vipA-1*). Infection with the strain carrying *Ptac*-*vipA^+^* led to a similar distribution of the effector as observed in JR32, although the protein was observed in the host cell earlier after uptake. This localization was lost in the *vipA-1* mutant, in which the effector was translocated in levels similar to the wild-type (Figure 6C) and homogeneously distributed in the host cell cytosol (Figures 6A and 7A).

In order to examine the colocalization of translocated VipA during the course of infection with endosomes and actin, infected cells were stained with respectively, an anti-EEA-1 antibody and rhodamine-phalloidin, a fluorescently-labeled protein that specifically binds F-actin. Colocalization was observed with both markers and increased over time, reaching the highest values at 8 hours post-infection (Figures 6B and 7A; data not shown). To determine the degree of colocalization with these two cell components, quantitative analysis was performed by calculating the Manders overlap coefficient, a commonly used approach for quantifying colocalization in fluorescence microscopy [19]. Briefly, in this experiment it corresponds to the fraction of blue pixels (VipA signal) that overlaps with green or red pixels (EEA-1/Early Endosome and Rhodamine-phalloidin/F-actin, respectively) (see Materials and Methods). As shown in Figure 7B, at 8 hr post-infection an average of 23% of VipA colocalized with EEA-1 and 40% with actin filaments, being these values approximately 10% higher in the case of the strain carrying *Ptac-vipA^+^*. Notably, in some cells more than 80% of VipA was associated with EEA-1 or actin (Figure 7B), often simultaneously with both (see also enlarged areas of Figure 7A), suggesting a dynamic interaction among these components.

We addressed the possible interference of VipA in host cell endocytic trafficking in several ways. Firstly, we looked for a defect in endocytic internalization of rhodamine-dextran in THP-1 macrophages previously infected with *L. pneumophila* wild-type or *ΔvipA*. In this experiment, we compared the total rhodamine fluorescence in both types of infected cells, and we investigated possible delays in trafficking of rhodamine-dextran by visualizing colocalization of this dye with anti-EEA-1 antibody over time. However, in neither case a significant difference was apparent, suggesting that endocytic internalization and early endosome trafficking is not being affected by the presence of VipA (data not shown). Additionally, the number and morphology of EEA-1 positive Early Endosomes were identical in these infected cells.

These observations demonstrate that during macrophage infection, translocated VipA binds actin filaments and early endosomes in the host cell. To our knowledge, no other currently identified Legionella effector displays either of these properties.

### VipA binding to cellular targets does not require additional Legionella effectors

The previous macrophage infection experiments did not exclude the possibility that additional Legionella effectors secreted during infection were required for the correct localization of VipA upon translocation into the host cell. Thus, in order to analyse VipA subcellular localization in the absence of other Legionella proteins, we expressed it ectopically in mammalian CHO cells and examined its association with microfilaments and early endosomes. For this purpose, a VipA-EGFP fusion protein was constructed and its expression placed under the control of the CMV promoter in a derivative of plasmid pEGFP-N1 (Clontech). The resulting plasmid was transfected into CHO FcγRII cells and these were fixed and processed after 48 hr. Similarly to what was observed in yeast and during infection, VipA-EGFP was distributed in puncta in transiently transfected CHO cells, whereas the mutant VipA-1-GFP showed homogeneous cytosolic distribution (Figure S2). To analyse its association with microfilaments, these cells were stained with Rhodamine-phalloidin. As shown in Figure S2, the degree of colocalization between VipA and actin was difficult to ascertain due to the large array of stress fibers present in CHO cells. Therefore, further visualization was made after the disassembly of stress fibers with Cytochalasin D, an actin depolymerizing agent [37], [38]. As observed in CHO cells expressing GFP, incubation with 10 µM Cytochalasin D disrupted the actin cytoskeleton, changing the organization of actin in the cell from a filament network to focal accumulations (Figure S2, bottom panel). The same actin reorganization occurred when VipA-GFP expressing cells were treated with cytochalasin, but interestingly the VipA puncta, which did not disassemble but appear to have coalesced into larger structures, clearly colocalized with the enduring small cortical actin foci (see colocalization plot in Figure S2). Actin rearrangements upon cytochalasin treatment were also observed in cells expressing the VipA-1 mutant fused to GFP, but the cytosolic distribution of VipA-1-GFP did not alter with cytochalasin treatment.

The localization of VipA-EGFP with early endosomes was also tested by immunofluorescence assays with an anti-EEA-1 antibody (Figure S3A). The degree of colocalization with this marker was on average approximately 35%, whereas in cells expressing the VipA-1 mutant the overlap was only 6% (Figure S3E; p<0.001, unpaired t test), and these values are similar to the ones obtained during infection (Figure 7).

VipA was shown in this study (see above) to colocalize with components of the yeast MVB pathway, namely Bro1 containing endosomes and the vacuole. The protein Alix (also known as AIP-1) is a component of the MVB and a human counterpart of yeast Bro1 and interestingly was shown to be involved in the assembly of microfilaments, constituting a novel link between the MVB and the actin cytoskeleton [39]. To investigate if VipA associated with other components of the endosomal/MVB pathway in addition to early endosomes, we carried out immunofluorescence experiments in transiently transfected CHO cells and tested colocalization of VipA-EGFP with LAMP-1 and Alix. As shown in Figures S3 C and D, some signal overlap was observed with both lysosomes and Alix. However, the average colocalization was relatively low (15%) and not distinguishable from the values obtained with the mutant form of VipA (Figure S3E). In addition, similar results were obtained when colocalization was tested with the ER marker KDEL (Figures S3B and E), ruling out an association with the secretory pathway.

Taken together, these observations demonstrate that VipA associates with cortical F-actin patches and early endosomes in mammalian cells, and this occurs independently of other secreted Legionella effectors. Moreover, ectopically expressed VipA-EGFP does not colocalize significantly with later components of the Endosomal/MVB Pathway, such as lysosomes or Alix, or with the ER.

## Discussion

The *L. pneumophila* effector VipA was identified in previous studies due to its ability to interfere with organelle trafficking in the yeast Multivesicular Body (MVB) Pathway [13]. In this work we have shown that VipA binds actin and is able to polymerize microfilaments *in vitro* without the requirement of additional bacterial or eukaryotic factors (Figure 3). During human macrophage infection, translocated VipA associates with actin filaments, as well as with early endosomes (Figure 7). This subcellular localization was also verified when VipA-GFP or VipA-mCherry were ectopically expressed in *S. cerevisiae* and mammalian CHO cells, showing further that this distribution is independent of other bacterial effectors (Figures 4, 5, S2 and S3). In addition, a mutation in VipA that abolished the interference of the protein in yeast vacuolar trafficking led simultaneously to a decreased affinity and ability to polymerize actin *in vitro* (Figures 2, 3) and the ability to associate specifically with the host cell targets (Figures 4–7, S2 and S3), indicating a function of VipA linking the actin cytoskeleton and the MVB pathway. Both these functions are new amongst the pool of *L. pneumophila* effectors characterized to date.

Many extra- and intracellular pathogens target host actin as a means to produce a successful infection. This often involves the hijacking of the Arp2/3 complex by recruiting or mimicking nucleation promoting factors (NPFs) by a diverse array of mechanisms that serve different purposes. Examples include the movement of *Listeria monocytogenes* and *Shigella* within the host cytosol and cell to cell spread by the formation of actin comet tails mediated by the effectors ActA [40] and IcsA, respectively [41], or the formation of actin pedestals in pathogenic *E. coli* EPEC and EHEC through the action of the effector Tir [42], [43]. However, the number of known bacterial effectors able to polymerize actin directly is reduced. *Salmonella enterica* serovar Typhimurium SipC nucleates actin with an efficiency identical to the Arp2/3 complex, and is also able to bundle and crosslink actin filaments [44], and in *Chlamydia* the protein TARP forms long unbranched actin filaments that similarly to SipC seem to facilitate the internalization of the pathogen [45]. Contrary to SipC and TARP, *Vibrio parahaemolyticus* VopL and VopF polymerize actin much more potently than activated Arp2/3, leading to the formation of stress fibers [46] or actin-rich filoform formation in infected cells, respectively [47]. More recently, *Rickettsia rickettsii* Sca2 was found to function as eukaryotic formin mimic and to be involved in actin tail formation [48]. Several observations support a role of VipA as an actin nucleator instead of alternative activities that would also increase actin polymerization, such as decreasing the actin critical concentration, increasing the elongation rate or severing microfilaments. In addition to decreasing the initial polymerization lag phase, VipA increases the actin polymerization rate, which would not happen if its effect was in decreasing the actin critical concentration (as is the case, for instance, of *Salmonella* SipA [49]; Figure 3A). The enhancement of actin polymerization is also not due to an effect on the elongation rate, which was observable but weak in assays with actin seeds (Figure 3D). Furthermore, a possible filament severing activity of VipA was not visible in Transmission Electron Microscopy experiments (data not shown) or assays with actin seeds.

When compared to the above mentioned bacterial and eukaryotic actin nucleators, His_6_-VipA has lower activity, which can be due to several reasons. One possibility is that the VipA protein used in our assays may not be fully active. In fact, like many actin-binding proteins, VipA may contain auto-regulatory regions that inhibit its activity or it may need to be activated by additional bacterial or eukaryotic factor(s) absent in our *in vitro* experiments. We cannot also dismiss the possibility that the presence of the histidine tag may be partially hindering the protein's function. Secondly, the unusually high number of effectors found in Legionella so far (>300) and their observed functional redundancy raises the possibility that additional effectors may act in concert with VipA in actin polymerization. Another explanation could be that the low activity of VipA is tailored to its predicted function in endosomal trafficking during infection. Rather than causing major alterations in the host cell microfilament network, as was proposed for VopL function, VipA may play a more subtle role where its activity is enough to allow interference with membrane traffic but not sufficient to disturb overall cell actin homeostasis.

The primary sequence of VipA contains a central coiled-coil region and a C-terminal proline-rich region (Figure 2A). Both motifs are typical mediators of interaction with other proteins, and Pro-rich regions in particular are involved in binding to profilin. However, no typical actin-binding motifs such as the WASP-homology 2 (WH2) domain or Formin-Homology domain 2 (FH2) are present (reviewed in [50], [51]). The absence of either of these actin-binding motifs in VipA raises the possibility of a novel molecular mechanism of actin assembly. The analysis of bacterial effectors that influence host cell actin dynamics has provided valuable information to understand how eukaryotic NPFs work, as many share similar modes of action and interacting partners. Thus, future functional studies of VipA may not only widen the knowledge concerning virulence factors targeting actin but may also contribute to a broader comprehension of actin dynamics in the eukaryotic cell.

Although present in all sequenced genomes of Legionella pneumophila strains (Philadelphia-1, Corby, Lens, Paris, Alcoy, 570-CO-H and 130b), similarly to most Legionella effectors studied to date VipA is neither required for intracellular replication nor uptake (Figure S1B; data not shown). However, *in vivo* colocalization experiments in yeast and mammalian cells provided some clues to its function in the eukaryotic cell. First, in both types of cells VipA forms punctate structures that localize in actin-rich regions. In yeast they often associate with the bud-neck, and with endosomal and cortical actin patches markers Abp1 and Abp140, respectively (Figure 4). In addition, VipA also colocalized with the MVB-associated protein Bro1 and the perivacuolar compartment observed in mutants defective in MVB formation that results from improper fusion of the endocytosed cargo with the vacuole (Figure 5). These observations are consistent with the fact that VipA causes defects in this pathway [13]. Similarly, during macrophage infection translocated VipA associates with host cell early endosomes and F-actin, often with the three components forming large agglomerates (Figure 7). Moreover, and in contrast to many characterized Icm/Dot substrates, the effector is not found in the LCV (Figure 6). To rule out the possibility that other T4SS substrates could mediate VipA subcellular localization during infection, we analysed its distribution when ectopically expressed in CHO cells and, therefore, in the absence other Legionella proteins. In concordance with the previous results, we observed VipA-EGFP association with cortical actin foci (Figure S2) and with the early endosome marker EEA-1, demonstrating that VipA does not require other bacterial factors to bind its eukaryotic targets. In addition, no significant colocalization was seen with later components of the Endosomal/MVB Pathway (MVB component Alix and the lysosome) or the Secretory Pathway (ER; Figure S3). The endocytic pathway is responsible for the uptake of particles and converges in early endosomes, where cargo sorting occurs and molecules targeted for degradation in lysosomes are internalized to intraluminal vesicles originating the MVB. This organelle subsequently travels to and fuses with late endosomes and eventually lysosomes. Formation of the MVB requires the Endosomal Complex Required for Transport (ESCRT), and proteins belonging to the Alix/Bro1 family are important components of the MVB, interacting with ESCRT components to control the fission and fusion events taking place in the endosome lumen in mammalian and yeast cells. In addition, Alix associates with actin and several actin-binding proteins and is involved in cytoskeleton assembly, being to date the only eukaryotic link between actin dynamics and MVB formation [52]. In this work, we found that Legionella VipA also connects the two processes. The wild-type protein binds actin and promotes growth of filaments *in vitro*, associates with host cell early endosomes and actin filaments and leads to defects in the MVB pathway when ectopically expressed in yeast. In contrast, the VipA-1 mutant is unable to bind or polymerize actin, no longer affects trafficking and displays a homogeneous cytosolic distribution. This shows that the ability of VipA to bind actin is related to its association with a specific subcellular location as well as its role in modulating organelle trafficking. In this context, our results are consistent with a model where VipA, through its dual function of regulating actin dynamics and binding to endosomal organelles, may play a role in altering vesicle trafficking in order to enable the pathogen to escape degradation. A possibility is a role in helping isolating the LCV from the endocytic pathway, a process mediated by Icm/Dot substrates [53]. This hypothesis is sustained by the observation of large agglomerates of endosomal vesicles, F-actin and VipA apart from the LCV during macrophage infection (Figure 7).

To our knowledge, VipA is the only bacterial effector that binds early endosomes and is implicated in actin dynamics in the host cell. Thus, further characterization of its mode of action will undoubtedly shed new light on the mechanisms employed not only by *L. pneumophila* but also by other pathogens to manipulate host cell pathways during infection.

## Supporting Information

Figure S1 **Effect of** ***vipA*** **in intracellular replication.** **A.** Immunoblot for VipA detection in cell extracts of *L. pneumophila* strains. Lane 1, strain KS79 (Δ*comR*); lane 2, LPIF3 (Δ*comR* Δ*vipA*); lane 3, 120 ng of purified His_6_-VipA. Equivalent amounts of cell extracts were loaded in lanes 1 and 2. Immunodetection was carried with a purified polyclonal antibody raised against His_6_-VipA. **B.** Infection of *A. castellanii* with *L. pneumophila* strains KS79, LPIF3 and *dotA* mutant carrying a plasmid containing a Ptac-GFP fusion (pXDC31; [58]). Infection was carried out at a Multiplicity of Infection (MOI) of 5. Replication was followed by time-course fluorescence measurements for 72 hours.
(TIF)

Figure S2 **Localization of VipA-GFP and VipA-1-GFP in transiently transfected CHO-FcγRII cells.** CHO-FcγRII cells were transfected with plasmids expressing EGFP (pEGFP-N1), VipA-EGFP (pIF203) or VipA-1-EGFP (pIF213). After 48 hours cells were fixed and actin filaments stained with Rhodamine-phalloidin 5 µg.ml^−1^ (top panel), or pre-incubated with Cytochalasin D 10 µM for 30 min (bottom panel) and subsequently fixed and stained. For the micrograph of VipA-EGFP and cytochalasin D, a plot is depicted showing the spatial distribution and intensity of GFP and Rhodamine signal intensities along the dashed line depicted. Representative images are shown.
(TIF)

Figure S3 **Colocalization of VipA-EGFP and VipA-1-EGFP with organelle markers in CHO-FcγRII cells.** CHO-FcγRII cells were transfected (see legend of Figure S2) and observed after staining with antibodies against: **A.** Early Endosomal (α-EEA-1); **B.** ER (α-KDEL); or **C.** Lysosome (α-LAMP-1) or **D.** α-Alix. **E.** The fraction of VipA-GFP or VipA-1-GFP colocalizing with each marker was quantified as described in legend of Figure 7 (see also Materials and Methods for details). Filled symbols represent cells expressing VipA-GFP, open symbols cells expressing VipA-1-GFP, and bars indicate mean and standard deviation. Statistical analysis was performed with unpaired t test, and p values obtained are indicated (**, p<0.01; ***, p<0.001). Representative enlarged regions of analysed cells (displayed in A, B, C and D) are shown below the graph.
(TIF)

Table S1 **Strains used in this study.**
(DOC)

Table S2 **Plasmids and oligonucleotides used in this study.**
(DOC)
