# Supplementary figures and images for: The Legionella pneumophila Effector VipA Is an Actin Nucleator That Alters Host Cell Organelle Trafficking
Source: PLoS Pathog. 2012 Feb 23;8(2):e1002546. doi: 10.1371/journal.ppat.1002546 (PMC3285593; doi:10.1371/journal.ppat.1002546)

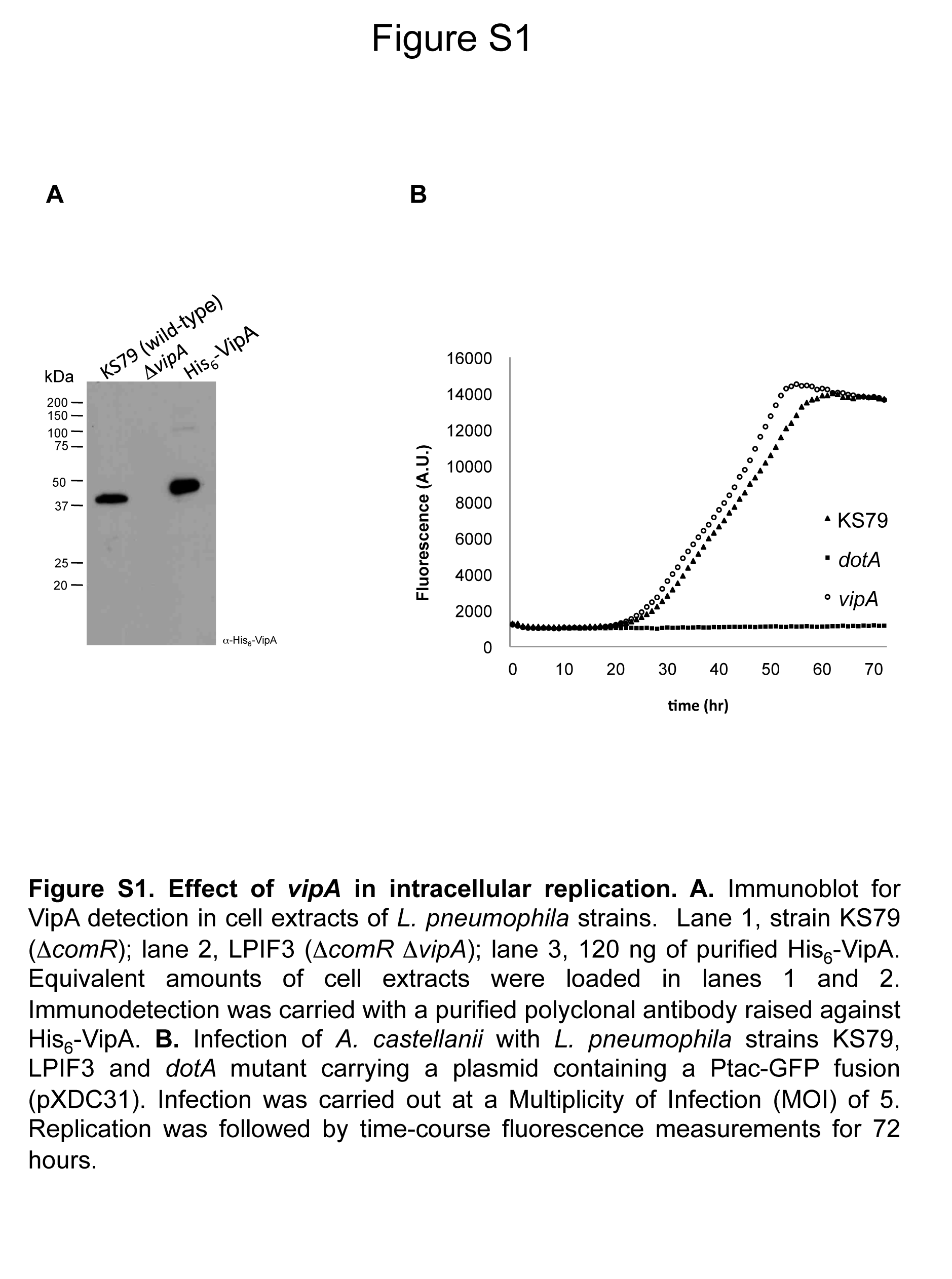

Supplement: Figure S1 — Effect of vipA in intracellular replication. A. Immunoblot for VipA detection in cell extracts of L. pneumophila strains. Lane 1, strain KS79 (ΔcomR); lane 2, LPIF3 (ΔcomR ΔvipA); lane 3, 120 ng of purified His6-VipA. Equivalent amounts of cell extracts were loaded in lanes 1 and 2. Immunodetection was carried with a purified polyclonal antibody raised against His6-VipA. B. Infection of A. castellanii with L. pneumophila strains KS79, LPIF3 and dotA mutant carrying a plasmid containing a Ptac-GFP fusion (pXDC31; [58]). Infection was carried out at a Multiplicity of Infection (MOI) of 5. Replication was followed by time-course fluorescence measurements for 72 hours. (TIF) [file ppat.1002546.s001.tif]

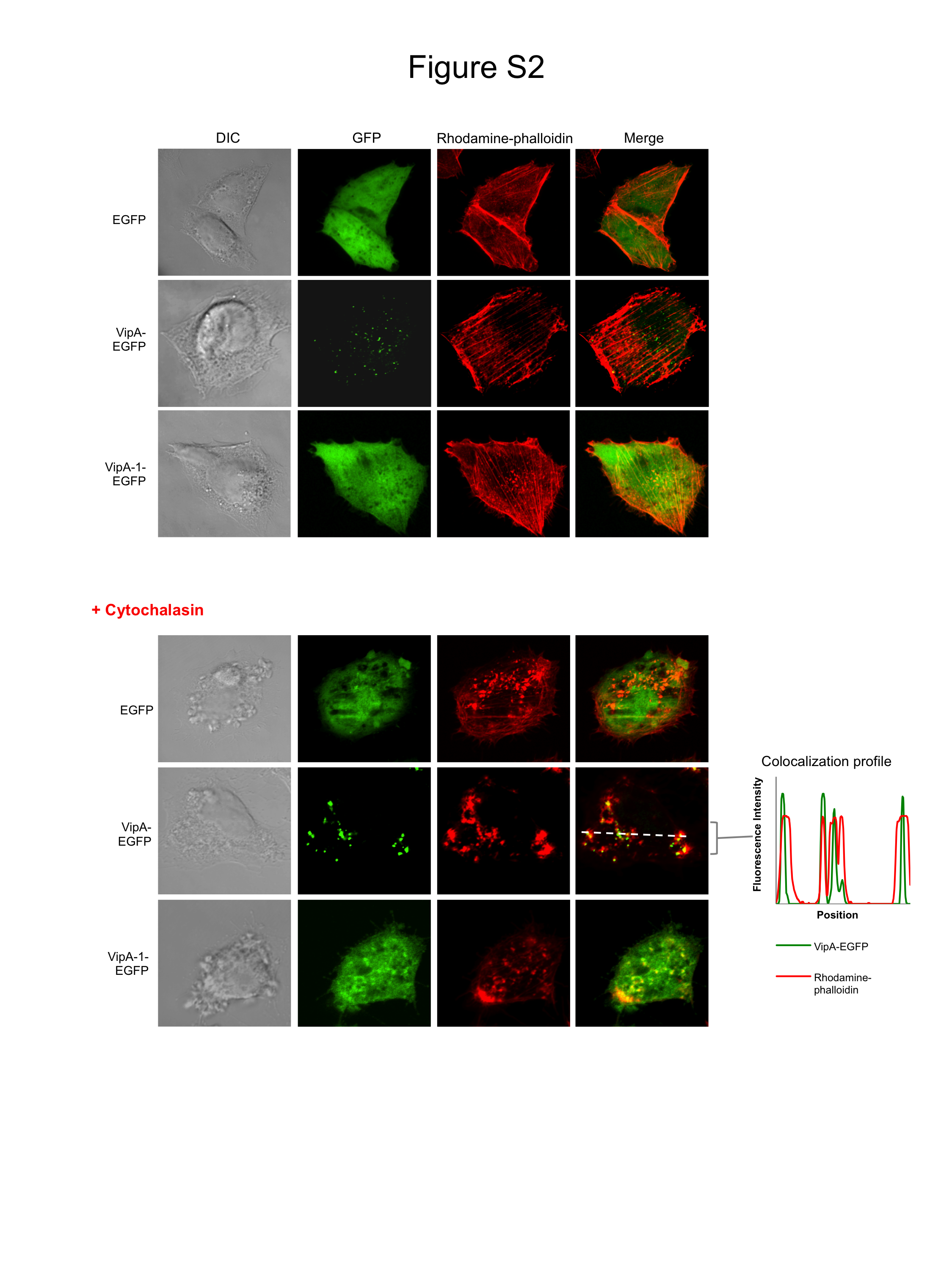

Supplement: Figure S2 — Localization of VipA-GFP and VipA-1-GFP in transiently transfected CHO-FcγRII cells. CHO-FcγRII cells were transfected with plasmids expressing EGFP (pEGFP-N1), VipA-EGFP (pIF203) or VipA-1-EGFP (pIF213). After 48 hours cells were fixed and actin filaments stained with Rhodamine-phalloidin 5 µg.ml−1 (top panel), or pre-incubated with Cytochalasin D 10 µM for 30 min (bottom panel) and subsequently fixed and stained. For the micrograph of VipA-EGFP and cytochalasin D, a plot is depicted showing the spatial distribution and intensity of GFP and Rhodamine signal intensities along the dashed line depicted. Representative images are shown. (TIF) [file ppat.1002546.s002.tif]

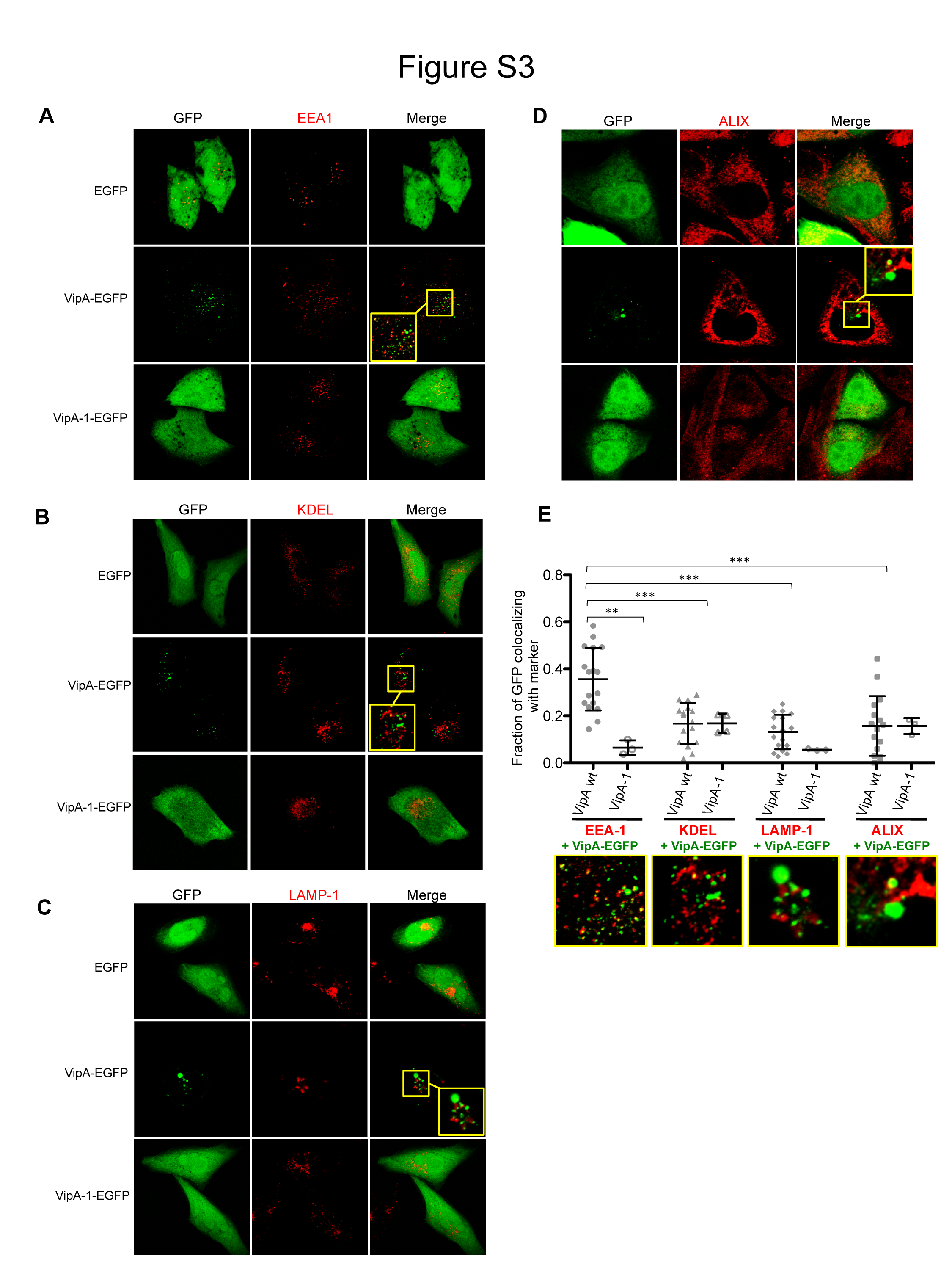

Supplement: Figure S3 — Colocalization of VipA-EGFP and VipA-1-EGFP with organelle markers in CHO-FcγRII cells. CHO-FcγRII cells were transfected (see legend of Figure S2) and observed after staining with antibodies against: A. Early Endosomal (α-EEA-1); B. ER (α-KDEL); or C. Lysosome (α-LAMP-1) or D. α-Alix. E. The fraction of VipA-GFP or VipA-1-GFP colocalizing with each marker was quantified as described in legend of Figure 7 (see also Materials and Methods for details). Filled symbols represent cells expressing VipA-GFP, open symbols cells expressing VipA-1-GFP, and bars indicate mean and standard deviation. Statistical analysis was performed with unpaired t test, and p values obtained are indicated (**, p<0.01; ***, p<0.001). Representative enlarged regions of analysed cells (displayed in A, B, C and D) are shown below the graph. (TIF) [file ppat.1002546.s003.tif]
