# Supplementary material for: The Legionella pneumophila Effector VipA Is an Actin Nucleator That Alters Host Cell Organelle Trafficking
Source: PLoS Pathog. 2012 Feb 23;8(2):e1002546. doi: 10.1371/journal.ppat.1002546 (PMC3285593; doi:10.1371/journal.ppat.1002546)
Supplement: Table S1 — Strains used in this study. (DOC) [file ppat.1002546.s004.doc]

**Table S**1. Strains used in this study

| **Strain** | **Relevant genotype** | **Construction*^a^*** | **Reference** |
| --- | --- | --- | --- |
| ***L. pneumophila*** |  |  |  |
| JR32 | Philadelphia-1, Sm^R^, r-m+ |  | [10] |
| KS79 | JR32 *ΔcomR* |  | [15] |
| LELA3118 | JR32 d*otA*::Tn*903*dll*lacz*3118 |  | [10] |
| LPIF3 | KS79 *ΔvipA::km^R^* |  | This study |
| LPIF11 | *ΔvipA* pMMB207c-*Ptac-vipA-1* | pMMB207c-*Ptac-vipA-1* → LPIF3 | This study |
| LPIF18 | *ΔvipA* pMMB207c-*Ptac-vipA* | pMMB207c-*Ptac-vipA* → LPIF3 | This study |
| ***S. cerevisiae*** |  |  |  |
| NSY01 | BHY10 diploid a/α, CPY-Inv, inv^-^, ura^+^ |  | [13] |
| SCIF00 | NSY01 *P_gal_-gfp* | pKS84 → NSY01 | [15] |
| SCIF01 | NSY01 *P_gal_-vipA-gfp* | pIF206 → NSY01 | This study |
| SCIF02 | NSY01 *P_gal_-vipA-1-GFP* | pIF209 → NSY01 | This study |
| THY157 | MAT*a his3Δ1 leu2Δ0 met15Δ0 ura3Δ0* *abp1:gfp:kanMX6* |  | [27] |
| YCY027 | MAT*a his3Δ1 leu2Δ0 met15Δ0 ura3Δ0* *abp140:gfp:kanMX6* |  | [27] |
| SCIF29 | *abp1-gfp P_gal_-mCherry* | pIF215 → THY157 | This study |
| SCIF30 | *abp1-gfp P_gal_ -vipA-mCherry* | pIF216 → THY157 | This study |
| SCIF31 | *abp1-gfp P_gal_ -vipA-1-mCherry* | pIF217 → THY157 | This study |
| SCIF32 | *abp140-gfp P_gal_ -mCherry* | pIF215 → YCY027 | This study |
| SCIF33 | *abp140-gfp P_gal_ -vipA-mCherry* | pIF216 → YCY027 | This study |
| SCIF34 | *abp140-gfp P_gal_ -vipA-1-mCherry* | pIF217 → YCY027 | This study |
| SCIF39 | *Bro1-gfp Pgal-mCherry* | pIF215→ JBY47 | This study |
| SCIF40 | *Bro1-gfp Pgal-vipA-mCherry* | pIF216→ JBY47 | This study |
| SCIF41 | *Bro1-gfp Pgal-vipA-1-mCherry* | pIF217→ JBY47 | This study |
| ***E. coli*** |  |  |  |
| NSEC45 | *E. coli* BL21 (DE3) pET15b-vipA |  | This study |
| NSEC55 | *E. coli* BL21 (DE3) pET15b-vipA-1 |  | This study |

***^a^*** The arrow indicates transformation and points from plasmid DNA to recipient strain.
