# Supplementary material for: The Legionella pneumophila Effector VipA Is an Actin Nucleator That Alters Host Cell Organelle Trafficking
Source: PLoS Pathog. 2012 Feb 23;8(2):e1002546. doi: 10.1371/journal.ppat.1002546 (PMC3285593; doi:10.1371/journal.ppat.1002546)
Supplement: Table S2 — Plasmids and oligonucleotides used in this study. (DOC) [file ppat.1002546.s005.doc]

**Table S**2. Plasmids and oligonucleotides used in this study

| **Plasmid** | **Relevant Characteristics** | **Reference** | |  |
| --- | --- | --- | --- | --- |
| pEGFP-N1 | Mammalian expression vector | Clontech | |  |
| pIF203 | pEGFP-N1 v*ipA* | This study | |  |
| pIF213 | pEGFP-N1 v*ipA-1* | This study | |  |
| pKS84 | *P_gal_*-*gfpURA3* | [15] | |  |
| pIF206 | pKS84 *P_gal_*-*vipA-gfp* | This study | |  |
| pIF209 | pKS84 *P_gal_*-*vipA-1-gfp* | This study | |  |
| pIF215 | *P_gal_*-*mCherry Leu2* | This study | |  |
| pIF216 | *P_gal_*-*vipA-mCherryLeu2* | This study | |  |
| pIF217 | *P_gal_-vipA-1-mCherry* | This study | |  |
| pET15b | *E. coli* expression vector | Novagen | |  |
| pET15b-vipA | pET15b *vipA* | This study | |  |
| pET15b-vipA-1 | pET15b *vipA-1* | This study | |  |
| pXDC31 | *Ptac-gfp* | [58] | |  |
| pMMB207c-vipA | *Ptac-vipA* | This study | |  |
| pMMB207c-vipA-1 | *Ptac-vipA-1* | This study | |  |
| **Oligonucleotide** | **Sequence (5’→3’)*^a^*** |  | |  |
| IF02 | TGGATCCCGGCGATTTTTTTTTTCGACGGG | |  | |
| IF03 | AACTCGAGCGCCACCATGGCGCCTATCAGTAATGCGTTTCTTAAG | |  | |
| IF04 | CGTTCTTGTTCATGAAATGGTGC | |  | |
| IF05 | GGAACTTCGAAGCAGCTCCAGCCTACACAATCCTCCTGATCCATGGAATCATAGTG | |  | |
| IF06 | GAACTAAGGAGGATATTCATATGGACCATGGCTTCTGTTCCTCCTGAATATAGGGC | |  | |
| IF07 | CGCATGCTGTGAATCTTTATTCCCTG | |  | |
| IF08 | GTTAGGATCCATGCCTATCAGTAATGCCTTTCTTAAG | |  | |
| IF09 | AGCTAAGCTTGAGATTTTTTTTTTCGACGGG | |  | |
| IF37 | AAAAGTCGACGCCTGACGCATATACCTTTT | |  | |
| IF38 | AAAACCCGGGGAACATATTCCATTTTG | |  | |
| IF39 | AAAAAAGCTTATGGTTTCCAAGGGC | |  | |
| IF40 | AAAACTGCAGGTCGACTCTAGATT | |  | |
| NSP22 | AGACATATGATGCCTATCAGTAATGCCTTT | |  | |
| NSP23 | AGAAGAGGATCCCTAGAGATTTTTTTTTTCGACGGG | |  | |
| NSP29 | AGAAGAGTCGACATGCCTATCAGTAATGCCTTTCTT | |  | |
| NSP30 | AGAAGAGTCGACCTAGAGATTTTTTTTTTCGACGGG | |  | |

*^a^* Restriction sites are underlined.
